# Psychrophily and Catalysis

**DOI:** 10.3390/biology2020719

**Published:** 2013-04-16

**Authors:** Charles Gerday

**Affiliations:** Laboratory of Biochemistry, Institute of Chemistry, University of Liege, Sart-Tilman, B-4000, Liege, Belgium; E-Mail: ch.gerday@ulg.ac.be; Tel.: +32-4366-3389; Fax: +32-43663364

**Keywords:** psychrophiles, cold-adapted enzymes, microcalorimetry, folding

## Abstract

Polar and other low temperature environments are characterized by a low content in energy and this factor has a strong incidence on living organisms which populate these rather common habitats. Indeed, low temperatures have a negative effect on ectothermic populations since they can affect their growth, reaction rates of biochemical reactions, membrane permeability, diffusion rates, action potentials, protein folding, nucleic acids dynamics and other temperature-dependent biochemical processes. Since the discovery that these ecosystems, contrary to what was initially expected, sustain a rather high density and broad diversity of living organisms, increasing efforts have been dedicated to the understanding of the molecular mechanisms involved in their successful adaptation to apparently unfavorable physical conditions. The first question that comes to mind is: How do these organisms compensate for the exponential decrease of reaction rate when temperature is lowered? As most of the chemical reactions that occur in living organisms are catalyzed by enzymes, the kinetic and thermodynamic properties of cold-adapted enzymes have been investigated. Presently, many crystallographic structures of these enzymes have been elucidated and allowed for a rather clear view of their adaptation to cold. They are characterized by a high specific activity at low and moderate temperatures and a rather low thermal stability, which induces a high flexibility that prevents the freezing effect of low temperatures on structure dynamics. These enzymes also display a low activation enthalpy that renders them less dependent on temperature fluctuations. This is accompanied by a larger negative value of the activation entropy, thus giving evidence of a more disordered ground state. Appropriate folding kinetics is apparently secured through a large expression of trigger factors and peptidyl–prolyl *cis*/*trans*-isomerases.

## 1. Introduction

Polar ecosystems are characterized by a high diversity and abundance of microorganisms. Indeed, cell densities from 0.9 to 14.9 × 10^5^ mL^−1^ have been recorded in Arctic pack ice with similar figures in adjacent seawater. Thirty-three phylotypes were identified in these environments; they belong to the γ- and α-proteobacteria with less than 1% being cultivable [1]. In Arctic tundra soils, and in winter, the total bacterial cell counts can be as high as 5 × 10^9^ cells per g of soil [2]. Even Arctic permafrost, characterized by temperatures below the freezing point of water, is highly populated in metabolically active or possibly dormant microorganisms with cell counts of 3.56 × 10^7^ per g of soil of Canadian permafrost [3]. High cell densities are also present in the Antarctic with figures of 5.4 to 7.9 × 10^7^ cells per g of lake sediment [4], whereas in free waters, at Terra Nova Bay (Ross Sea) for example, cell counts vary from 0.1 to 15.7 × 10^5^ cells mL^−1^ [5]. These cell densities, both in the Arctic and Antarctic oceans, are similar to those recorded in temperate habitats and correspond to microbial diversities much greater than those initially expected [6]. Such data testify to successful adaptations of microbial communities to extremely cold environments. Knowing that low temperatures have usually a negative effect on population growth, one has to conclude that a complete set of molecular adaptations has taken place to notably compensate for the freezing effect of low temperatures on reaction rate, diffusion rate, membrane permeability and nucleic acids dynamics, for instance. Reaction rates are clearly crucial for the survival of microorganisms at low temperatures, since they vary in an exponential way as a function of temperature according to the Arrhenius law, in which the rate constant, k = A. e^−Ea/RT^, depends on the pre-exponential factor A, also called frequency factor, which, in the reaction rate expression, derived from the transition state theory, takes the form of A = *k_B_*(T/*h*)exp(+ΔS^*^/R)exp(1); *k_B_* is the Boltzmann constant, *h*, the Planck constant, T, the temperature in Kelvin and ΔS^*^ the activation entropy of the reaction [7]. One can see that the frequency factor A strongly depends on the activation entropy and is directly dependent on temperature. E_a_ is called the activation energy; it is equal to the term, ΔH* + RT, and the activation enthalpy of the reaction can be easily determined from Arrhenius plots in which lnk is expressed as a function of 1/T. In enzyme-catalyzed reactions, these Arrhenius plots usually give a straight line of slope—E_a_/R over a more or less wide range of temperatures provided that the temperature conditions do not alter the enzyme structure or the enzyme–substrate complex. In the case of psychrophiles, one has also to take into consideration the viscosity of the environment which can have a strong effect on reaction rates. In 2004, Garcia-Viloca and coworkers [8] proposed, in the case of enzyme-catalyzed reactions, a generalized expression of reaction rate, in which k_cat_ = *γ*_(T)_*k_B_*(T/*h*)exp(−ΔG^*^/RT). The factor *γ* is, in the context of viscosity, an extended expression of the old transmission factor κ that takes into account the probability that some of the activated molecules will return to the ground state rather than be transformed into product; in other words, they can re-cross the energy barrier. This factor is usually neglected, but at low temperature, it can significantly differ from unity*.* Some works have been devoted to this problem and we can mention that of Demchenko *et al.* [9] who studied the influence of the viscosity of the medium on the catalytic properties of lactate dehydrogenase. They, for example, demonstrated that the V_max_, for lactate oxidation in the presence of NAD^+^, decreases from 8.5 units in low-viscosity buffer to 1.5 in a 44% sucrose solution equivalent to a viscosity of about 6cP. It is worth noting that at 20 °C the average viscosity of the intracellular space is 2.5cP, whereas at 0 °C, this viscosity raises to 5cP [10]. Also, if at 20 °C the viscosity of pure water is close to 1, it subsequently raises to 1.787 at 0 °C. Thus, clearly the high viscosity of aqueous media at low temperatures should also have a depressive effect on reaction rates. This problem has been also addressed by Siddiqui and coworkers [11].

## 2. General Properties of Cold-Adapted Enzymes

**Figure 1:**
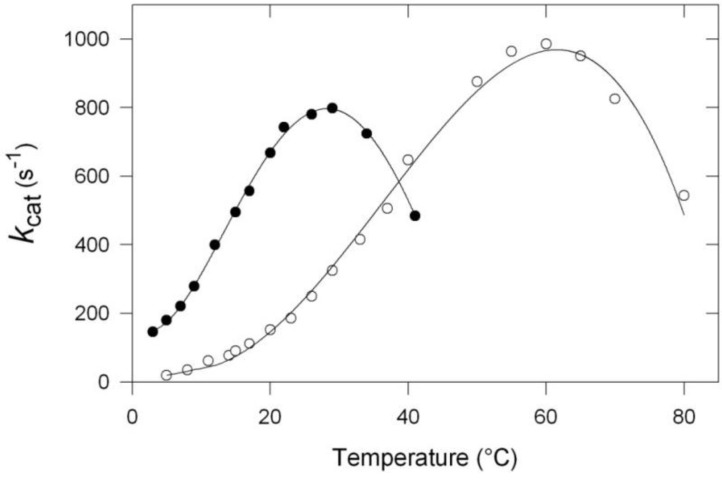
Specific activity as a function of temperature of the α-amylase from the Antarctic strain *Pseudoalteromonas haloplanktis* (black dots) and of the thermophilic counterpart from *Bacillus amyloliquefaciens* (open circles). Worth noting is the important shift of the apparent optimum towards low temperature. Adapted from [12].

Many cold-adapted enzymes have now been fully characterized in terms of catalytic properties and the main characteristic of these enzymes is that the thermo-dependent activity curve is always displaced towards low temperatures as illustrated in Figure 1. The left curve corresponds to the evolution of the activity as a function of temperature of a cold-adapted α-amylase from the Antarctic strain *Pseudoalteromonas haloplanktis.* The right curve illustrates a similar curve recorded for the homologous α-amylase from a thermophilic microorganism, *Bacillus amyloliquefaciens* [12]. Three main differences can be observed:

(1). The apparent optimum temperature of the cold-adapted enzyme is displaced towards low temperatures by as much as 30 °C.
(2). The cold-adapted enzyme displays a much higher catalytic efficiency than the thermophilic enzyme up to approximately its apparent optimum.
(3). The cold-adapted enzyme is, in contrast with the thermophilic one, rapidly inactivated at temperatures above 25 °C.

Some additional commentaries are necessary to fully appreciate the significance of these curves. First, one has to consider that the so-called “optimum temperature” still reported as such in many papers, is only an apparent optimum since, at this temperature, the enzyme is already under severe thermal stress and cannot be exposed at this temperature for a long time. A more appropriate term would be “critical temperature,” to indicate that a partial inactivation has already taken place. Second, as far as the shift towards low temperature is concerned, it is worth mentioning that its amplitude strongly depends on the enzyme investigated. Third, in the case of many cold-adapted enzymes, the activity that corresponds to the apparent optimum is somewhat lower than that recorded for homologous mesophilic or thermophilic enzymes, and this can possibly reflect an incomplete adaptation to low temperatures. Also, in the same context, the higher activity, observed at low and moderate temperatures, considerably varies with the enzyme under investigation. In the present cases, at 10 °C, the specific activity of the cold α-amylase is about 10 times as high as that of the thermophilic enzyme.

The first significant report on the properties of cold-adapted enzymes was made in 1984 and concerned a heat labile alkaline phosphatase isolated from Antarctic seawater bacteria [13]. Not only the three main properties of cold-adapted enzymes were correctly described, but the authors also suggested that these enzymes could present significant advantages over mesophilic counterparts for biotechnological purposes. Recent investigations devoted to the study of new cold-adapted enzymes have strictly confirmed the properties of these enzymes. We can mention the hormone-sensitive lipase isolated from the Antarctic strain *Psychrobacter* sp. TA144 [14], the periplasmic nitrate reductase from the Antarctic bacterium *Shewanella gelidimarina* [15], and the serine hydroxymethyltransferase from a cold-adapted *Psychromonas ingramii* isolated from Arctic polar sea ice [16]. Another parameter which can be influenced by temperature is the K_m_ which, in general, is related to the affinity of the enzyme for the substrate, provided that the rate constants that could interfere with the constants directly involved in the true dissociation constant of the enzyme–susbtrate complex could be neglected. The comparison of the K_m_ values of enzymes from orthologous species, differently adapted to temperature (Table 1), reveals three main features:

**Table 1:** K_m_ values of some psychrophilic, mesophilic and thermophilic enzymes.

Enzyme-organism	T (°C)	K_m_	Ref
**Alpha-amylase**			
P: *Pseudoalteromonas haloplanktis*	25	234.00 uM	[17]
M: Pig pancreatic	25	65.00	
**Aspartate aminotransferase**			
P: *P. haloplanktis*	07	5.82 mM	[18]
	25	8.34	
M: *E.coli*	25	7.31	
	35	21.04	
**Aspartate transcarbamylase**			
P: Gram-TAD1	11	20.00 mM	[19]
M*: E.coli*	30	0.014	
**Citrate synthase**			
P: Antarctic bacterium DS2-3R	23	230 uM	[20]
M: mesophiles		<50 uM	
**DNA ligase**			
P: *Pseudoalteromonas haloplanktis*	04	0.165 uM	[21]
M*: E.coli*	18	0.179	
T: *Thermus scotoductus*	18	0.179	
	30	0.702	
T: *Thermus scotoductus*	45	0.236	
**Elongation factor TU**			
P: *Moraxella* TAC II 25	15	0.36 uM	[22]
M*: E.coli*	15	0.13	
**Endonuclease I**			
P: *Vibrio salmonicido*	05	246.00 mM	[23]
M: *Vibrio cholerae*	05	118.00	
**Isocitrate dehydrogenase**			
P: *Colwellia maris*	15	62.00 mM	[24]
M: *E. coli*	15	3.30	
**Lactate dehydrogenase**			
P: *Champsocephalus gunnarii*	00	0.16 mM	[25]
M: *Deinococcus radiodurans*	48	0.21	
T: *Thermus thermophilus*	90	0.16	
**Ornithine transcarbamylase**			
P: *Moritella abyssi*	05	1.78 mM	[26]
M: *E. coli*	37	2.40	
T: *Thermus thermophilus*	55	0.10	
**RNA-dependent ATPase**			
P: *Pseudoalteromonas haloplanktis*	10	0.60 mM	[27]
	25	0.9	
M: *E.coli*	10	0.02	
	25	0.06	
**Subtilisin**			
P: *Bacillus* (Antarctic)	05	26.00 uM	[28]
	25	37.00	
M: *Bacillus licheniformis*	05	6.00	
	25	17.00	
**Triose phosphate isomerase**			
P: *Vibrio marinus*	10	1.90 mM	[29]
M: *E. coli*	25	1.09	

First, an increase in the temperature over a threshold value causes an increase in K_m_; in other words, a lower affinity of the enzyme for the substrate; second, the lowest K_m_ values are observed in the temperature range usually experimented by the organism [30] third, with two exceptions, aspartate aminotransferase and DNA ligase, the K_m_ values of cold-adapted enzymes are usually higher than those of mesophilic counterparts but are closer to each other at the respective temperature of their environment. These data suggest that enzymes from species adapted to different temperatures have evolved molecular adaptations in order to maintain a conformation enabling an appropriate interaction between enzymes and substrates at the usual temperature of the environment. In certain cases, these adaptations are not likely to be complete, as previously mentioned.

## 3. Activity and Stability

**Figure 2:**
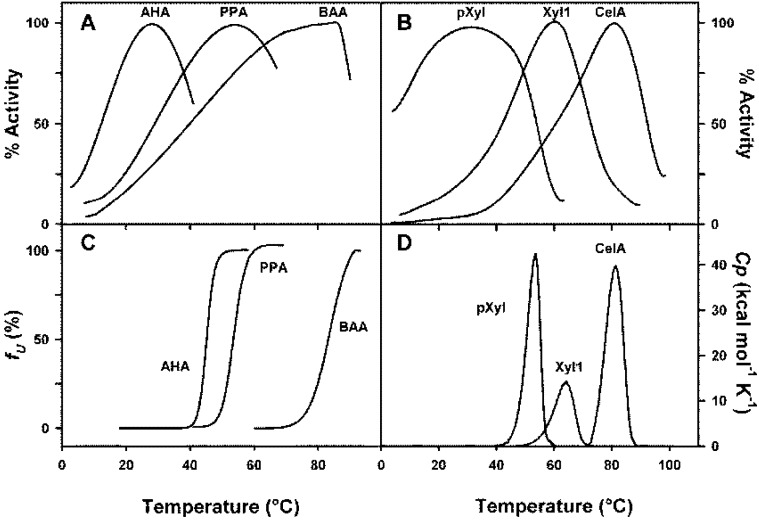
**(A)** Percentages of specific activities of psychrophilic (AHA), mesophilic (PPA) and thermophilic (BAA) α-amylases as a function of temperature and **(C)** concomitant thermal transitions as observed by fluorescence spectroscopy. **(B)**. Percentages of specific activities as a function of temperature of psychrophilic xylanase from the Antarctic strain *Pseudoalteromonas haloplanktis* (pXyl), mesophilic xylanase from *Streptomyces* sp. S38 (Xyl 1), and thermophilic endoglucanase from *Clostridium thermocellum* (Cel A) and their **(D)** concomitant thermal unfolding as recorded by differential scanning calorimetry. Reproduced with permission from [40].

Various techniques have been used to evaluate the thermal stability of proteins. The thermal unfolding of a protein is accompanied by a positive modification of the enthalpy and the heat absorption can be followed in a microcalorimeter that directly provides the Tm values of the respective domains of a protein or, if the unfolding is highly cooperative, the Tm value of the whole molecular edifice. Fluorescence signals can also be used because the exposure to the solvent of tryptophane residues, usually buried into the protein, is associated with a red shift of the emission wavelength, which is easily followed as a function of temperature. Circular dichroism signals are also altered as a function of unfolding; in some cases, the far U-V region has been used, but in this range of wavelength, around 220 nm, only the secondary structure is concerned and the ellipticity values are related to the percentage of helical and beta-structures. This does not allow the detection of the modifications of the tertiary structure, which can be evaluated only if the circular dichroïsm measurements are carried out in the near U-V region, in the absorption bands of aromatic residues. The signals are, however, rather weak, and the ellipticity changes can be positive or negative upon unfolding. In many cases, also the thermal unfolding has been determined using the measurement of the residual activity of the enzyme after exposure for a certain time at a given temperature followed by cooling. This technique is not suitable due to the possible refolding on cooling. To validate this technique, one has first to demonstrate that the thermal unfolding is truly irreversible at all temperatures tested. In this context, it is worth knowing that psychrophilic enzymes are particularly prone to rapid spontaneous refolding. One of the characteristic features of cold-adapted enzymes is that, often, the thermal inactivation of the enzyme precedes any detectable changes in tertiary structure by the techniques mentioned above. This is clearly illustrated in Figure 2. This figure shows the profiles of the thermal inactivation and structural transition curves of orthologous α-amylases (Figure 2A,C) and glycoside hydrolases, xylanases and cellulase, (Figure 2B,D) adapted to different temperatures. In the case of α-amylases, the thermal unfolding has been followed by fluorescence spectroscopy at an emission wavelength of 350 nm, and one can see that for the cold-adapted enzyme (AHA) a significant inactivation is reached before any detectable changes in the three-dimensional structure of the protein. On the contrary, in the case of mesophilic and thermophilic enzymes, the inactivation strictly corresponds to detectable changes in the three-dimensional structure. Similar data are observed in the case of psychrophilic, mesophilic and thermophilic glycoside hydrolases (Figure 2B,D). It is worth mentioning that thermograms in panel 2D have been obtained by differential scanning calorimetry. They correspond to the measure of the heat absorbed during the thermal unfolding of the proteins. These data suggest either that the active site of these psychrophilic enzymes are more heat-labile than the protein structure or that the substrate-enzyme complex becomes highly unstable and that its thermal dissociation precedes any change in the structure of the protein. This hypothesis is supported by the fact that the K_m_ values of cold-adapted enzymes are, in general, higher than that of their mesophilic or thermophilic counterparts. For these latter, the loss of activity is concomitant with unfolding. The higher specific activity of cold-adapted enzymes, at low and moderate temperatures, can be attributed to activation energies lower than those of their mesophilic and thermophilic counterparts, as shown in Table 2. This lower activation energy is the result of a drastic reduction of the activation enthalpy. This corresponds to a lower temperature dependence of the activity and suggests that less enthalpy-driven bonds have to be broken to secure an appropriate interaction between the enzyme and the substrate which, in general, is an induced process. The Eyring equation, k_cat_=κ. k_B_T/h. e^-ΔG*/RT^ which is another form of the Arrhenius equation, and in which κ is the transmission coefficient; k_B_, the Boltzmann constant; h, the Planck constant and ΔG*, the free energy of activation, indicates that the catalytic constant is not only exponentially dependent on temperature, but also on the free activation energy. Table 2 shows that this free energy of activation is, as expected from the values of the activation enthalpies, lower than that of the mesophilic counterparts, with two exceptions: arginine kinase and chitinase. The small amplitude of the differences between the respective activation energies is the result of larger negative values of the activation entropies in the case of cold-adapted enzymes that act as a compensating factor. Indeed, the differences observed in the catalytic constants would have been much larger if similar values had been recorded for the activation entropies of psychrophilic and mesophilic enzymes. The more negative values of the activation entropies of cold-adapted enzymes also suggest that the ground state of these enzymes displays a larger disorder than their mesophilic homologues. As mentioned earlier, there are two exceptions to these observations: arginine kinase and chitinase, in these cases, the activation energy is apparently higher than those of the mesophilic counterpart, despite the fact that both the activation enthalpy and activation entropy strictly follow the usual trend of psychrophilic enzymes. As ΔG* derives from the difference between the enthalpic and entropic terms, it is probable that the higher activation energy of the cold-adapted arginine kinase is due to experimental errors on these terms. In the case of chitinase, the situation is possibly different since it has been argued that the lower specific activity of this psychrophilic enzyme at 15 °C originates from the fact that a soluble preparation of chitin from crabs was used as substrate and this may not be a good substrate for the cold-adapted enzyme, since chitins from different origins can be structurally very different [31]. The analysis of Table 2 also shows that, in some cases, the activation entropy is positive, meaning that the activated state displays a higher disorder than the ground state. This is only recorded in the case of mesophilic enzymes and can be either attributed to a particularly high rigidity of these mesophilic enzymes or, alternatively, to a difference in the redistribution of water molecules associated with the enzyme. Although the differences observed in the free energy of activation could seem rather weak, they are however high enough to explain the higher specific activity of cold-adapted enzymes. Indeed, and as an example, the difference in the activation energy of mesophilic and psychrophilic α-amylases is only of 0.8kJ/mole at 10 °C but this is enough to secure a threefold increase of the k_cat_ for the cold-adapted enzyme.

**Table 2:** Catalytic constants and activation parameters of a few cold-adapted enzymes as compared with mesophilic counterparts.

Enzyme	Type	T (°C)	k_cat_(s^−1^)	ΔG*	ΔH*	TΔS*	Reference
				kJ/mole			
Amylase	Psy	10	294.0	57.7	34.7	−23.0	[32]
	Mes		97.0	58.5	46.4	−12.1	
Arginine kinase	Psy	25	3.3	69.4	18.8	−50.6	[33]
	Mes		13.4	66.6	41.9	−4.7	
Cellulase	Psy	4	0.18	71.6	46.2	−25.4	[34]
	Mes		0.01	78.2	65.8	−12.4	
Chitinase	Psy	15	1.7	69.2	60.2	−9.0	[31]
	Mes		3.9	67.2	74.3	+7.1	
Chitobiase	Psy	15	3.8	59.5	44.7	−14.8	[35]
	Mes		0.9	63.5	71.5	+8.0	
Citrate synthase	Psy				7.4	ΔS* = 22.7	[20]
	Mes				11.5	ΔS* = 9.7	
Endonuclease	Psy	5	9.41	62.8	33.4	−29.4	[23]
	Mes		1.03	67.9	74.0	+6.1	
LDH	Psy	0	250.0	75.0	22.0	−53.0	[25]
	Mes		72.0	75.0	45.0	−30.0	
Lysozyme	Psy			45.1	31.9	−13.2	[36]
	Mes			46.2	49.4	+3.2	
Subtilisin	Psy	15	25.4	62.0	36.0	−26.5	[37]
	Mes		5.4	66.0	46.0	−20.2	
Xylanase (bact)	Psy	10	515.5	54.0	21.0	−33.0	[38]
	Mes		59.5	60.0	58.0	−2.0	
Xylanase (yeast)	Psy	5	14.8	52.3	45.3	−7.0	[39]
	Mes		4.9	54.6	49.9	−4.7	

## 4. Thermodynamic Stability

The thermodynamic stability of a protein can be easily evaluated at a given temperature by differential scanning calorimetry provided that the unfolded form is in a two-state reversible thermodynamic equilibrium with the folded structure according to the equation: N ⇌ U, defined by a thermodynamic equilibrium constant K. Then, the Gibbs free energy of unfolding, also known as thermodynamic stability, can be calculated from the Gibbs–Helmholtz equation: ΔG_N-U_ = ΔH_N-U_ − TΔS_N-U_ = −RTln K. This equation can be rewritten as a function of the information derived from a differential scanning calorimetry profile, as shown in Figure 2, panel D.


ΔG_N-U(T)_ = ΔH_cal_ (1 − T/T_m_) + ΔC_p_(T − T_m_) − TΔC_p_ln(T/T_m_)

**Figure 3:**
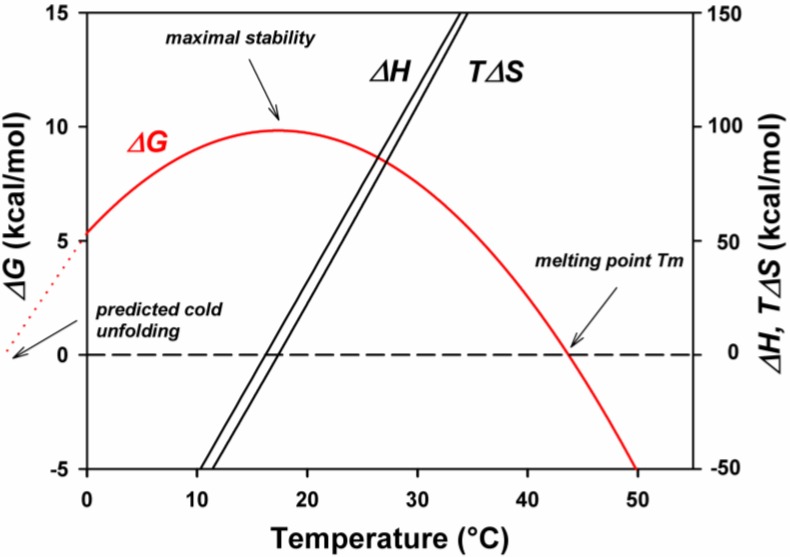
A conformational stability curve displaying the change in stabilization energy, ΔG, as a function of temperature. It also shows the concomitant change of the enthalpic, ΔH, and entropic, ΔS, contributions. It is worth noting that the relatively small values of ΔG result from the difference between rather large figures of ΔH and TΔS. Reproduced with permission from [44].

**Figure 4:**
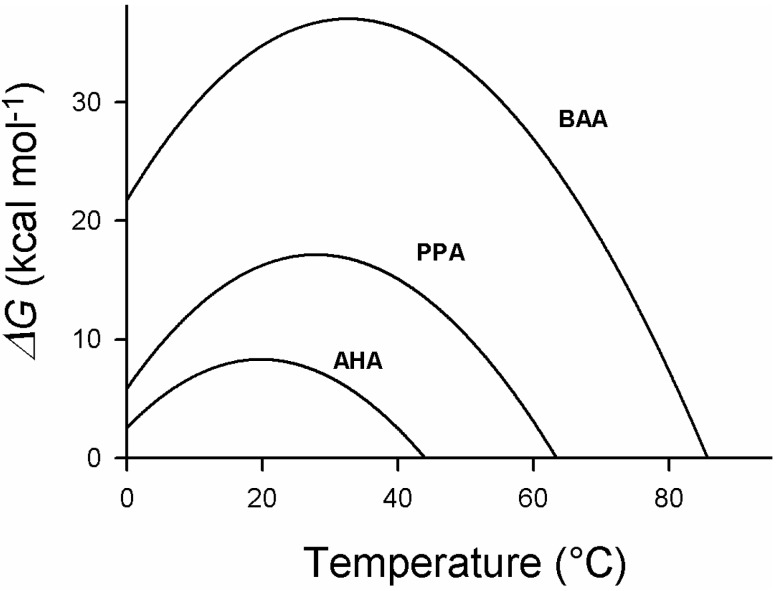
Stabilization energy, ΔG, of psychrophilic (AHA), mesophilic (PPA) and thermophilic (BAA) α-amylases as a function of temperature. Note that the maximum stability is for the three enzymes close to each other, around 25 °C, despite the large difference in the melting temperatures of these enzymes. AHA (*Pseudoalteromonas haloplanktis* α-amylase); PPA (Pig pancreatic α-amylase); BAA (*Bacillus amyloliquefaciens* α-amylase). Adapted from [32].

ΔH_cal_, is the heat absorbed on unfolding; it is given by the area limited by the curve; T is the temperature investigated; T_m_ is the temperature of half-unfolding for ΔG_N-U_ = 0 when U/N = 1; ΔC_p_, is the heat capacity change from the native to the unfolded state and is mainly due to the exposure of hydrophobic groups followed by their hydration [41,42]. It corresponds to the amount of surface exposed to the solvent upon unfolding, in other words, to the accessible surface area (ASA). This factor can also be determined experimentally using a microcalorimeter or can be calculated if the three-dimensional structure is known; it is a positive value. A typical thermodynamic stability curve is shown in Figure 3, in which are also described the thermodynamic components of the above mentioned equation. One can see that the maximum thermodynamic stability is obtained around 20 °C, the enthalpic and entropic terms vary with the temperature and one can predict an unfolding induced by cold around −10 °C. Experimentally, such stability curves have been obtained in the case of psychrophilic, mesophilic and thermophilic α-amylases, as shown in Figure 4. Several interesting features can be deduced from the analysis of these curves. First of all, the maximum stability, which corresponds to the highest value of ΔG_N-U,_ is for the three orthologous enzymes recorded around 25 °C, even so, the melting temperatures are very different, around 42 °C for AHA, 62 °C for the mesophilic enzyme, and 85 °C in the case of the thermophilic α-amylase. This has been attributed to the hydrophobic effect that plays a crucial role in the folding and stability of proteins [43]. Second, in the case of mesophilic (PPA) and thermophilic enzymes (BAA), the usual environmental temperature of these organisms lies on the right limb of the stability curve and therefore does not correspond to the maximum stability of these enzymes. This low stability at the environmental temperature is in fact required to secure an appropriate flexibility of the molecular edifice that allows a good interaction of these enzymes with the substrates. This flexibility is secured through the increase of temperature of the unfavorable stabilization entropy, ΔS_N-U_. At the maximum stability, ΔS_N-U_ = 0, this term becomes negative at low temperatures due to the propensity of hydrophobic groups to favor hydration rather than association with similar groups.

Therefore, on the right side of the curve, mesophilic and thermophilic enzymes are stabilized by the enthalpic term. Conversely, in the case of the cold-adapted enzyme, the environmental temperature lies on the left side of the curve; this part of the curve is characterized by a negative value of the entropic term, which is therefore the stabilizing factor. The negative value of the stabilization enthalpy, chiefly the result of a weakening of hydrophobic and electrostatic interactions induced by the hydration of these groups, appears, on the other hand, to be essential in conferring the appropriate flexibility of the psychrophilic enzyme at low temperatures.

Ultimately, the hydration of these groups leads to cold denaturation and, paradoxically, the cold-adapted enzyme is more sensitive to low temperatures than the homologous mesophilic and thermophilic enzymes. It will be shown that this is due to the fact that the structure of the cold-adapted enzyme is stabilized by a lower number of interactions. In the case of the orthologous α-amylases, one can see that the higher stability of the mesophilic and thermophilic enzymes, leading to higher melting points, is obtained by lifting the stability curve, that is, by increasing the amplitude of the enthalpic contribution to the stability [32]. This is mainly achieved through reinforcing hydrophobic interactions and increasing the number of hydrogen and ionic interactions next to a non-systematic decrease of the entropic contribution. This latter factor can result from an increase in the number of proline residues and a decrease in the number of glycine residues that both decrease the entropy of the denatured form. As a consequence, notably of the decrease of hydrophobic interactions in cold-adapted enzymes, many of them show a reversible thermal unfolding due to their lower propensity to form aggregates when the hydrophobic core is exposed to the solvent.

## 5. Engineering Cold-Adapted Enzymes

About thirty-three dimensional structures of cold-adapted enzymes have been solved by X-ray crystallography at high resolution (Table 3) and the analysis of these structures has largely contributed to defining the structural determinants of the lower stability of these enzymes. As already stated, these factors include: a lowering of the strength and/or number of the usual weak interactions; the hydrophobic, ionic and hydrogen bonds; a higher proportion of glycine residues, providing an increase of the mobility of certain regions; a decrease in the number of proline residues, also acting in this direction (there is, indeed, a tendency toward an increase in proline residues from psychrophiles to thermophiles); the weakening of the hydrophobic clusters that contributes to reduce the compactness of the protein; an increase in the number of hydrophobic residues at the surface of the molecule (this process induces a clustering of water molecules around these groups and induces a decrease in the entropy and a concomitant increase in free energy); the increase of the number of charged groups at the surface that favors the interaction with the solvent and the solubility at low temperatures; the substitution of the N- and C-caps of α-helices by uncharged residues that reduce the interactions between these residues and the dipoles formed by the helices that indeed carry a net positive charge at the N-terminal part and a net negative charge near the C-terminal end [45]; the increase of the length of the loops connecting the secondary structures; a decrease in the metal-binding affinity in metalloproteins; and, finally, psychrophilic proteins can exhibit loose protein extremities, which are preferentially tightly bound or buried in thermophilic proteins. Each protein adapted to cold uses a few of these factors in various combinations for the adaptation, and this seriously complicates the identification of the factors that are truly involved, even when the three-dimensional structure is known. A first approach to shed some light on the molecular characteristics of cold adaptation is site-directed mutagenesis. Amino acids substitutions can be introduced on the basis of hypotheses resulting from the analysis of crystallographic or of model structures of these enzymes. This rational approach is rendered hazardous by the fact that, in the absence of thermal selective pressure, some genetic drift probably occurs that tends to give rise to structural modifications not really implicated in the adaptation to cold. In one of the pioneering studies [17], numerous single mutations were introduced in the cold-adapted α-amylase isolated from the Antarctic strain *Pseudoalteromonas haloplanktis.* This enzyme displays a severe reduction of stability when compared to mesophilic and thermophilic counterparts (Figure 4). The effect of the mutations, supposed to restore structural specificities found in mesophilic and thermophilic counterparts, were evaluated both at the level of thermal stability and specific activity. Fourteen mutations, including double mutants, were introduced to reproduce weak bonds found in the highly similar structure of the homologous mesophilic enzyme from pig which displays eight additional ion pairs, fifteen additional arginine residues, and ten additional aromatic interactions. The introduced mutations aimed to restore hydrogen bonds, salt bridges, helix dipole stabilization, and various hydrophobic interactions. All these mutations were located far from the catalytic site. Some interesting conclusions were drawn: the additional selected electrostatic interactions provided the highest contribution to stability, increasing both the Tm and ΔH_cal_, the latter by values as high as 4.4 kcal/mol at 20 °C; a double mutant, consisting in an additional salt bridge and double aromatic interactions, increased the stabilization energy of the cold-adapted enzyme by a factor of two at 10 °C; and, the reinforcement of the hydrophobic core structure induced the appearance of multiple calorimetric domains absent in the cold-adapted enzyme characterized by a two-state unfolding process. Stabilized mutants were also found to refold with less efficiency, and most of the mutations that provided a mesophilic character by increasing the stability of the cold-adapted enzyme also led to a decrease in both k_cat_ and K_m_. This can be explained by the reduction of the conformational entropy of the binding species. It was also concluded that the cold-adapted enzyme has reached a limit in thermal stability precluding any further improvement of the specific activity through a decrease in stability. In another similar work carried out on this enzyme, an additional disulfide bond was also introduced to mimic the situation occurring not only in pig pancreatic α-amylase, but also in all chloride-dependent α-amylases from mammals and birds [46]. In these organisms, this disulfide bridge connects domains A and B and is located near the active site. As expected from the previous study, the introduction of this disulfide bridge decreases the specific activity by a factor higher than two, as well as the K_m_ both at 5 °C and 20 °C. The mutant also shows a higher compactness as demonstrated by fluorescence-quenching experiments, whereas its microcalorimetric characterization displays, contrarily to the wild cold-adapted enzyme, a profile typical of the pig pancreatic enzyme with multiple transitions. However, the T_m_ of the first transition was lower than that of the cold-adapted enzyme. This is indicative of an unfavorable constraint created by the introduction of the disulfide bridge. Stabilizing effects are, however, recorded in other parts of the enzyme since the overall calorimetric enthalpy increases from 214 kcal mol^−1^ for the cold-adapted enzyme to 241 kcal mol^−1^ for the mutant CC. These values have to be compared to 295 kcal mol^−1^ found for the pig pancreatic α-amylase. In a recent work, two multiple mutants of this cold-adapted α- amylase, derived from the data recorded from single mutations, were also investigated [47]. The first, Mut5, bears five mutations, previously described, that correspond, as stated above, to structural peculiarities existing in the mesophilic pig pancreatic α-amylase: N150D introduces a salt bridge, V196F restores an aromatic interaction, K300R provides a bidentate interaction with the chloride ion, and T232V reinforces a hydrophobic cluster, as well as Q164I. The second mutant, Mut5CC, is identical to Mut5 with the addition of the disulfide bridge Q58C/A99C found in chloride dependent α-amylases from mammals and birds. First of all, the specificity of the two multiple mutants, towards various natural and chromogenic substrates, was strictly similar to that of the parent cold-adapted enzyme. By contrast, the mutants showed a specific activity similar to that of the mesophilic enzyme and a very significant increase of the thermal stability as recorded by fluorescence spectroscopy and differential scanning calorimetry. Contrarily again to the mesophilic enzyme, the thermal inactivation above 35 °C occurs without any detectable structural changes supporting the idea that the active site remains the most thermal sensitive structural element. Fluorescence quenching, using acrylamide as quencher of the tryptophane residues, shows that Mut5 and Mut5CC display a reduced structural permeability. The reversibility of the unfolding was also strongly affected and the rate constants for irreversible unfolding differed by several order of magnitude at 43 °C. Reversible unfolding was only possible in the presence of non-detergent sulfobetaine, 3-(1-pyridinio)-1-propane-sulfonate. It was concluded that these two mutants, as well as the single mutants from which they derive, can be considered as structural intermediates between the psychrophilic and the mesophilic enzymes. These data also strongly supports the prevailing hypothesis that the reduction of the force of the weak interactions that stabilize the cold-adapted enzyme induces an increase in the flexibility of the enzyme. This provides an appropriate accessibility of the substrate at low temperature that leads to a higher specific activity of cold-adapted enzymes at low and moderate temperatures to the expense of thermal stability. This view was further supported by a recent and independent work related to the molecular dynamics of these mutants. The aim of this study was to identify in atomic details the effect of these mutations, especially the long-distance effects, and to identify the structural determinants that led to the incomplete conversion of the cold-adapted enzyme into a mesophilic-like edifice [48]. Multiple MD simulations were applied to the seven mutants (single and multiple) and it was shown that these mutants display a reduced flexibility in various regions of the protein, especially near the active site and substrate-binding groove. They also elicit, in some cases, unexpected long-range effects that even affect the flexibility of domain C that did not carry any mutation. This confirms the ability of these mutations to modulate the dynamic properties of AHA in conferring to it a mesophilic-like behavior, both in terms of activity and substrate binding. The current view of the relationship between enzyme dynamics, flexibility, and catalytic properties was contested in a study that submitted a cold-adapted protease to directed evolution [49]. After several cycles, a multiple mutant of the cold-adapted subtilisin S41 [28] was generated associating a high thermostability to a high specific activity at low temperatures. The selection of the mutants was made on the basis of a higher stability coupled with a specific activity higher or equal to that of the wild-type enzyme towards a synthetic substrate, s-AAPF-pNa. It was concluded that, at least *in vitro*, it was quite possible to simultaneously increase the stability of an enzyme concomitantly with its specific activity. The fact that, in nature, these two parameters seem to evolve in opposite directions could be due to the absence of selective pressure on thermal stability in low temperature environments. Later, however, it was reported that the mutant was poorly active towards macromolecular substrates. The importance of the choice of the substrate in this type of experiment making use of multi-substrates enzymes was underlined in similar experiments tending to confer to a mesophilic subtilisin BPN^’^ a cold-adapted character. Indeed, mutants associating a reduced stability to a higher specific activity were obtained only when synthetic substrates were used but not when casein, a natural substrate, was used [50]. Other attempts to confer a higher activity at low temperature were carried out on a thermophilic protease, WF146, showing a high proportion (>60%) of identical amino acids with the aforementioned cold-adapted protease [51]. Here again, a combination of random and site-directed mutagenesis was used using casein as substrate. The selected mutants were found to be more active than the wild-type enzyme and were all less stable, especially the mutant R29 that includes four single mutations. It had a specific activity at 25 °C, three times as high as that of the wild-type enzyme and showed a half-life of 4.5 min at 80 °C to be compared to 60 min for the wild-type enzyme. In this experiment, it was also shown that this multiple mutant and the other mutants were found to be less active than the wild-type enzyme over the synthetic substrate s-AAPF-pNA and displayed higher Km. This confirms that, generally, the high specific activity of cold-adapted enzymes is gained to the detriment of the thermal stability. This is necessary to confer an appropriate flexibility of crucial parts of the molecular structure at low temperature that facilitates the accommodation of the substrate and probably also the release of products. The difference in the catalytic properties of the afore-mentioned mutants towards low and high molecular weight substrates reflects this trend. A mutant well adapted to small-sized substrates will be too rigid to properly interact with larger substrates and, conversely, mutants well adapted to large-sized substrates will be relatively less efficient in the case of small-sized substrates due to an excess of flexibility reflected in a Km increase, as observed previously. That does not necessarily mean that, *in vitro*, it is not possible to both increase, to a certain extent, the thermal stability and specific activity of a cold-adapted enzyme. Nature has not probably tested all the possibilities and has evolved enzymes up to the point where, in a specific environment, an appropriate compromise between stability–flexibility and activity was reached [44].

**Table 3:** Crystallographic structures of cold-adapted enzymes obtained at high resolution.

Cold-Adapted Enzyme	Host Organism	Reference
Adenylate kinase	*Bacillus globisporus*	[52]
Adenylate kinase	*Marinibacillus marinus*	[53]
Alkaline metalloprotease	*Pseudomonas* sp.	[54]
Alkaline phosphatase	*Pandalus borealis*	[55]
Alkaline phosphatase	*Bacterial strain* TAB5	[56]
Anionic trypsin	*Salmo salar*	[57]
Alpha-amylase	*Pseudoalteromonas haloplanktis*	[58]
Aspartate carbamoyltransferase	*Moritella profunda*	[59]
Beta-galactosidase	*Arthrobacter* sp. C2-2	[60]
Catalase	*Vibrio salmonicida*	[61]
Cellulase	*Pseudoalteromonas haloplanktis*	[62]
Citrate synthase	*Arthrobacter* sp. strain DS2-3R	[63]
Elastase	*Salmo salar*	[64]
Endonuclease I	*Vibrio salmonicida*	[23]
Esterase	*Pseudoalteromonas* sp. 643A	[65]
Lactate dehydrogenase	*Champsocephalus gunnari*	[25]
Lipase B	*Candida antarctica*	[66]
Malate dehydrogenase	*Aquaspirillium articum*	[67]
Pepsin	*Gadus morhua*	[68]
Phenylalanine hydroxylase	*Colwellia psychrerythtaea*	[69]
Proteinase K-like	*Serratia* sp.	[70]
Protein-tyrosinephosphatase	*Shewanella* sp.	[71]
S-formylglutathione hydrolase	*Pseudoalteromonas haloplanktis*	[72]
Subtilisin-like protease	*Vibrio* sp. PA-44	[73]
Subtilisin S41	*Bacillus* sp.	[74]
Superoxide dismutase	*Aliivibrio salmonicida*	[75]
Triose phosphate isomerase	*Vibrio marinus*	[29]
Trypsin	*Oncorhynchus ketav*	[76]
Uracil-DNA N-glycosylase	*Gadus morhua*	[77]
Xylanase	*Pseudoalteromonas* sp.	[78]

## 6. Folding at Low Temperature

**Figure 5:**
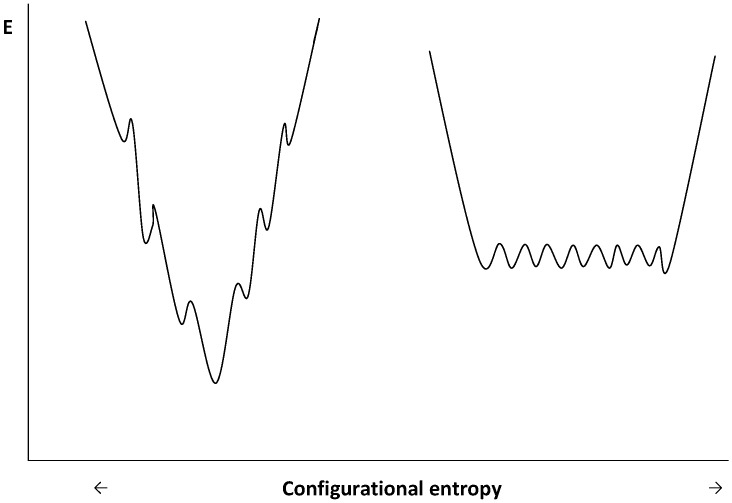
Schematic representation of funnel-shaped folding energy landscapes of mesophilic (left diagram) and psychrophilic (right diagram) enzymes. Adapted from [32].

Protein synthesis consists in the production, at the ribosome level, of unfolded polypeptide chainsthat should either properly fold in the complex environment of the intracellular space or be conditioned in order to safely reach their final destination, in organites, cellular envelopes, or extracellular space. Although some proteins fold spontaneously without problem, others need assistance by proteins known as chaperones. These, indeed, help the protein to adopt a favorable equilibrium between the unfolded and folded state; they assist the protein up to their specific localization, prevent misfolding, aggregation, and actively participate in the appropriate turnover by controlling the hydrolysis of non-functional proteins. They also play an active role in the assembly of multimeric proteins [79,80]. Psychrophilic, mesophilic and thermophilic proteins presumably fold according to similar but very complex processes that involve the nucleation-condensation mechanism and/or parallel routes. The first mechanism occurs through the production of native-like secondary structures, involving residues separated by short sequences, stabilized by hydrogen bonds that induce a condensation of the structure around this nucleus and the formation of intermediate tertiary structures stabilized by other bonds and characterized by various transition state barriers [81]. The second mechanism, known as parallel routes, involves different folding channels in which misfolding is a natural consequence of hierarchical folding and that productive folding intermediates are also stabilized by non-native interactions [82]. The folding process can be viewed as a folding funnel populated by various and transient energy-distinct intermediates converging in a more or less defined native form characterized by a lower free energy level. Psychrophilic and mesophilic–thermophilic proteins, however, display distinct folding funnels as proposed by D’Amico *et al.* [32] in the case of cold-adapted α-amylases. This is illustrated in Figure 5. As cold-adapted enzymes are less stable than their mesophilic counterparts, the average energy level of the native state is higher than that of the mesophilic counterpart, whereas the difference in dynamical properties is reflected by the number of local minima and in their possible inter-conversion between each other on the free energy landscape. In this context, one can see that the native state of the cold-adapted enzyme is characterized by a large population of conformations differing by a low energy level, phenomenon that allows the rapid conversion of one conformation into another. This can explain the high structural flexibility of psychrophilic enzymes. By contrast, in the mesophilic counterpart, the number of local free energy minima is limited. They display quite distinct energy levels that limit the conversion between the conformations represented on the conformational coordinates. That explains the lower flexibility of mesophilic enzymes at a given temperature. This model was found to be consistent with a cold-adapted zinc metalloprotease studied by molecular dynamics [83] and by similar comparative studies on the molecular dynamics of cold-adapted and mesophilic elastases and uracil DNA glycosylases [84]. It was concluded that psychrophilic enzymes present a greater number of metastable states at relevant temperatures and can explore several conformational basins that favor the activity at low temperatures, whereas mesophilic enzymes tend to be trapped into the main conformational basin. The lower energy level of the unfolded form of psychrophilic enzymes reflects an increase in the entropy of the unfolded form of some psychrophilic enzymes when compared to their mesophilic counterparts due to their higher proportion of glycine residues and to the reduction of their number of proline residues. It has already been mentioned that low temperatures, in favoring the hydration of individual groups, weaken some intra-molecular interactions, such as hydrophobic and ionic interactions, and also counteract the proper conversion of the *trans* configuration of proline residues into a *cis* configuration, a process which is a crucial rate-limiting step in the folding of proteins .These facts can be detrimental to the correct folding of proteins at low temperature, and it is essential to understand how folding is regulated in psychrophiles. From the limited number of studies devoted to this problem that make use of the differential expression of proteins at various growth temperatures, one can conclude that psychrophiles, although they are able to express all the chaperones discovered in mesophilic counterparts, fail to adopt a generalized strategy. Furthermore, depending on the species, even contrasting data were recorded. For example, in *Oleispira antarctica*, it was shown that the main chaperones GroEL and GroES had an optimum refolding activity at low temperature and were also found, by contrast to those of *E. coli*, to confer to the mesophilic bacterium the ability to grow at low temperatures [85,86]. However, in *Methanococcus burtonii* [87], as well as in the Antarctic bacterium *Pseudoalteromonas haloplanktis* [88], the expression of GroEL was strongly repressed and, by contrast, overexpressed in *Sphingopyxis alaskensis* [89] and *Acidithiobacillus ferrooxidans* [90]. For those microorganisms that repress, at low temperatures, the expression of the main chaperone GroEL, it has been hypothesized that, in psychrophiles, the hydrophobic core is often weaker than in mesophilic counterparts and, therefore, the risk of aggregation and misfolding is rather limited and the expression of GroEL is not generally required. This is supported by the fact that many cold-adapted proteins display a reversible thermal unfolding [44]. Also, in *E. coli,* the overexpression of the trigger factor at low temperature represses the expression of other chaperones [91]. The discrepancy observed in these data can possibly originate from the choice of the cardinal temperatures selected for comparison. Some of them correspond to the so-called optimum growth temperature based on the shortest doubling time, and it is well known that these temperatures induce a severe thermal stress in bacteria. This can significantly modify the data. There is, however, a common phenomenon observed at low temperatures; it is the systematic overexpression of the peptidyl–prolyl cis/trans isomerase and of the trigger factor which was found to embark a PPIase domain [92]. This clearly indicates that the *cis-trans* isomerization of prolyl residues is a particularly significant limiting step of the folding process of proteins at low temperatures.

## 7. Conclusions

Life at low temperature requires the expression of enzymes that can compensate for the freezing effect of these temperatures on molecular structures. Indeed, cold can prevent the appropriate interaction with the substrate and is exponentially detrimental to the rate of the chemical reactions occurring in living organisms. Psychrophiles, during the course of evolution, have slightly altered the amino acid sequence of their enzymes in order to produce molecular structures characterized by a higher flexibility than their mesophilic counterparts at low temperatures. This was achieved through a decrease of the strength and number of classical weak interactions such as hydrogen and ionic bonds, as well as hydrophobic interactions, but also by an increase of the entropy of the unfolded state through a decrease in proline residues and an increase in glycine residues. Depending on the enzyme and on its capacity to tolerate some structural modifications, only a few of these destabilizing factors are mobilized so that the strategy in the adaptation appears specific to each enzyme. This has led to a spectacular increase of the specific activity of these enzymes at low and moderate temperatures that reaches more or less that of their mesophilic counterparts at their usual environmental temperatures. This strategy has been, however, generally detrimental to their thermal stability, which is drastically depressed with no consequence for these organisms as no selective pressure on thermal stability is exerted in their low temperature environments. Depending on their specific evolution history and on the physico–chemical characteristics of their permanent habitats each psychrophile has developed on this basis a specific strategy that led to a sort of continuum in the adaptation to cold of these enzymes. Indeed, some of these organisms are able to tolerate temperatures around 40 °C, while others display more accentuated characteristics with a limit of survival below 15 °C. The folding process of proteins is strongly dependent on temperature, and the production of cold-adapted enzymes necessitates some adjustments at this level. In this case also, the strategy adopted by cold-adapted organisms seems to be specific with a modulated expression of the various chaperones that assist folding. However, one consistency seems to be the overexpression of factors that facilitate the conversion of *cis* form of proline residues into their *trans* conformers, such as the enzyme peptidyl–prolyl *cis/trans* isomerase and the related trigger factor.
